# Primary Skull Base Lymphoma Presenting With Ipsilateral Abducens Nerve Palsy: A Case Report

**DOI:** 10.7759/cureus.73108

**Published:** 2024-11-06

**Authors:** Alexander Schuster Bruce, Victoria Grammatopoulou

**Affiliations:** 1 Otolaryngology, Royal Surrey County Hospital, Guildford, GBR

**Keywords:** abducens nerve palsy, diffuse large b-cell lymphoma, lymphoma, matrix combination chemotherapy, mri, parosmia, primary skull base lymphoma, skull base, sphenoid sinus tumour

## Abstract

A 66-year-old woman with no prior medical history presented to the emergency department with diplopia and parosmia. The neurological examination identified an isolated left abducens nerve (CN VI) palsy. A head CT scan, followed by a brain MRI, showed a large, locally advanced tumour in the left sphenoid sinus with extensive skull base involvement and perineural extension into the left orbit. The histopathological analysis of the sphenoid sinus biopsy revealed high-grade diffuse large B-cell lymphoma. A PET scan confirmed this was a primary bone lymphoma. MRI of the pituitary, without contrast, six weeks following the initial imaging showed a more extensive local tumour extension. The patient was initially treated with MATRIX chemotherapy and targeted therapy. This case report describes a rare presentation of primary skull base lymphoma with abducens nerve palsy.

## Introduction

Primary non-Hodgkin’s lymphoma of the bone (PLB) is an uncommon malignancy, accounting for just 1%-2% of all malignant lymphomas [1]. The majority of PLB cases involve the pelvis and limbs, while primary skull base lymphomas (PSBLs) are exceptionally rare. A 2017 review identified only 21 cases of PSBL reported between 1992 and 2015 [2]. Even more infrequently, PSBL presents as a unilateral abducens nerve palsy, with only a limited number of cases reported in the literature [3,4].

## Case presentation

A 66-year-old woman presented to the emergency department (ED) with a six-day history of intermittent diplopia on lateral gaze. She reported recent parosmia, for which her general practitioner had prescribed clarithromycin, suspecting sinusitis. On neurological examination, an isolated unilateral left abducens nerve (CN VI) palsy was noted, with no other neurological abnormalities observed. She denied any history of epistaxis or prior sinus issues and reported feeling well systemically, with no fever, weight loss or nocturnal sweating. No lymphadenopathy was identified, and blood tests, including haematology, were unremarkable. She had no significant medical history and was not taking any regular medications.

**Figure 1:**
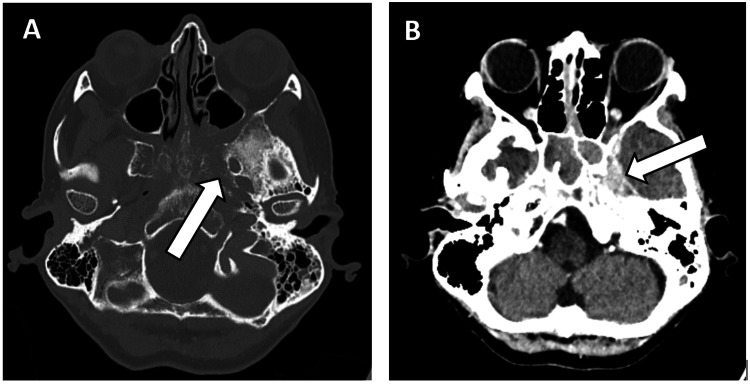
A head CT scan demonstrating a dense material within the left sphenoid sinus, with erosion of the wall of the left sphenoid sinus and adjacent sphenoid bone (A). Additionally, there is a high-density collection/enhancing soft tissue in the left Meckel's cave (B), adjacent to the eroded left sphenoid bone.

**Figure 2:**
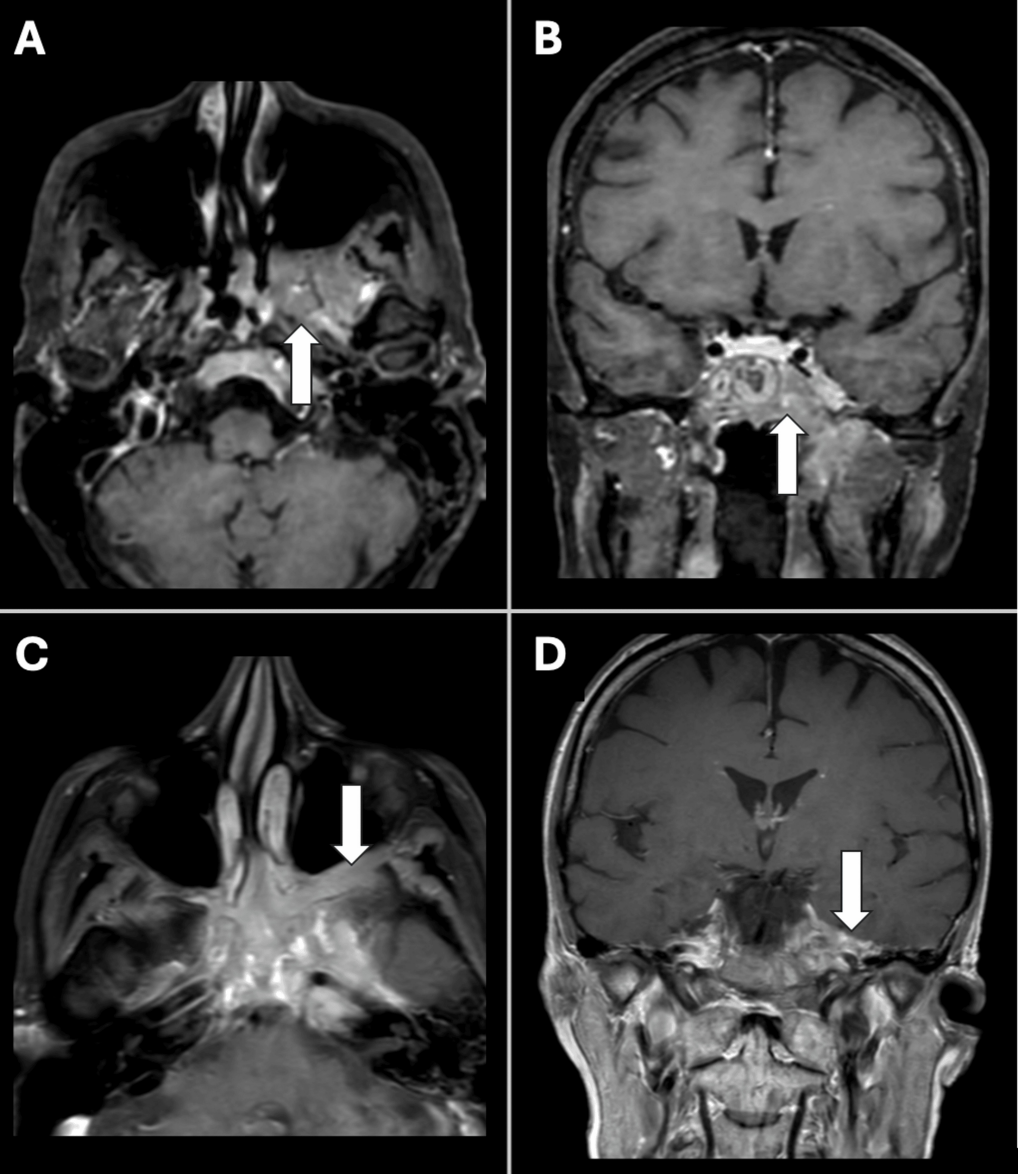
Initial contrast-enhanced MRI of the head T1-W images demonstrating an enhancing mass centered on the body of the left sphenoid bone (A, B). Extensive soft tissue perineural extension is observed, obliterating the left pterygopalatine fossa and extending anterolaterally along the posterior wall of the maxillary antrum (C). Additionally, there is direct invasion through the skull base with dural infiltration along the medial floor of the middle cranial fossa (D).

Intravenous ceftriaxone was initiated in the ED due to a suspected worsening sinus infection, in conjunction with steroid and decongestant sprays. A head CT scan revealed a dense material within the left sphenoid sinus, extending to the adjacent sphenoid bone and clivus. Additionally, a high-density enhancing soft tissue lesion was observed in the Meckel’s cave, adjacent to the eroded left sphenoid bone (Figure 1). The following day, an MRI of the head and sinuses, with contrast, revealed a large, locally advanced lesion in the left sphenoid sinus, with direct extension into the skull base and left orbit, as well as dural infiltration along the medial floor of the middle cranial fossa (Figure 2). 

A diagnostic endoscopic sinus surgery was performed following the MRI, with tumour biopsies taken from the left superior turbinate ridge and both left and right sphenoid sinuses. The tumour appeared white and soft, without macroscopic evidence of necrosis. Thick secretions were noted in the right sphenoid sinus due to tumour obstruction of the drainage pathway. Access to the left sphenoid sinus was challenging due to significant osteitis and vascularity. 

**Figure 3:**
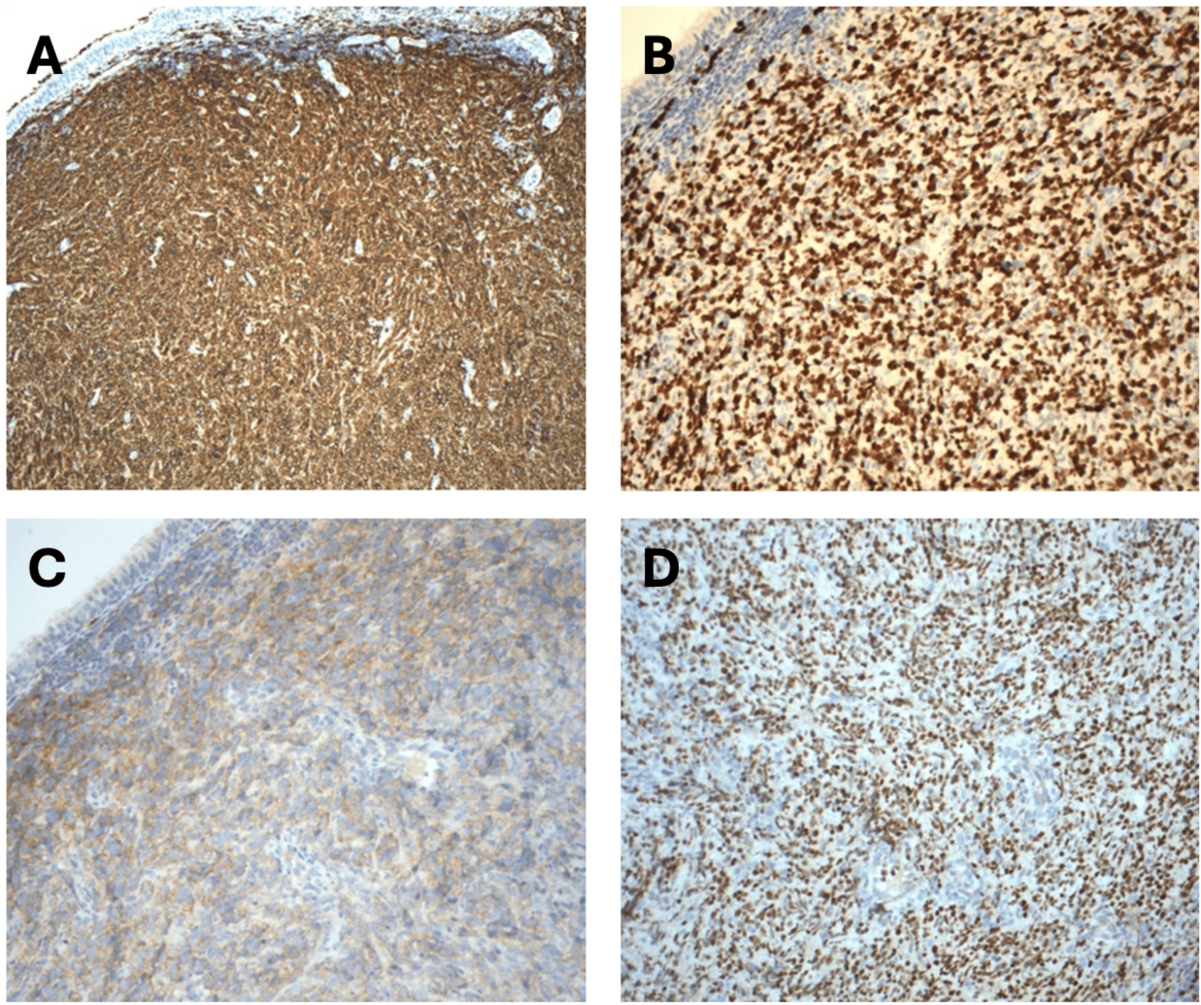
Microscopic analysis of the tumour revealing characteristics of high-grade diffuse large B-cell lymphoma (DLBCL), an aggressive lymphoma arising from the B-cells within the lymph nodes. Specific histological markers confirm this diagnosis: tumour cells are positive for CD20 (A), a surface marker that identifies B-cells; BCL6 (B), a protein commonly present in germinal center B-cells, indicating the tumour’s origin within this area of the lymph node; and CD10 (C), another germinal center marker, appearing weakly in this case. A high proliferation rate of 95% (D) demonstrates rapid cell division, highlighting the tumour’s aggressive nature.

The histopathology showed tumour cells positive for CD20 and BCL6 with an MIB1 proliferative index of 95%, consistent with a diagnosis of high-grade diffuse large B-cell lymphoma (DLBCL) of the germinal center type (Figure 3). A subsequent PET scan revealed no FDG uptake, confirming that the sphenoid tumour was a primary malignancy.

**Figure 4:**
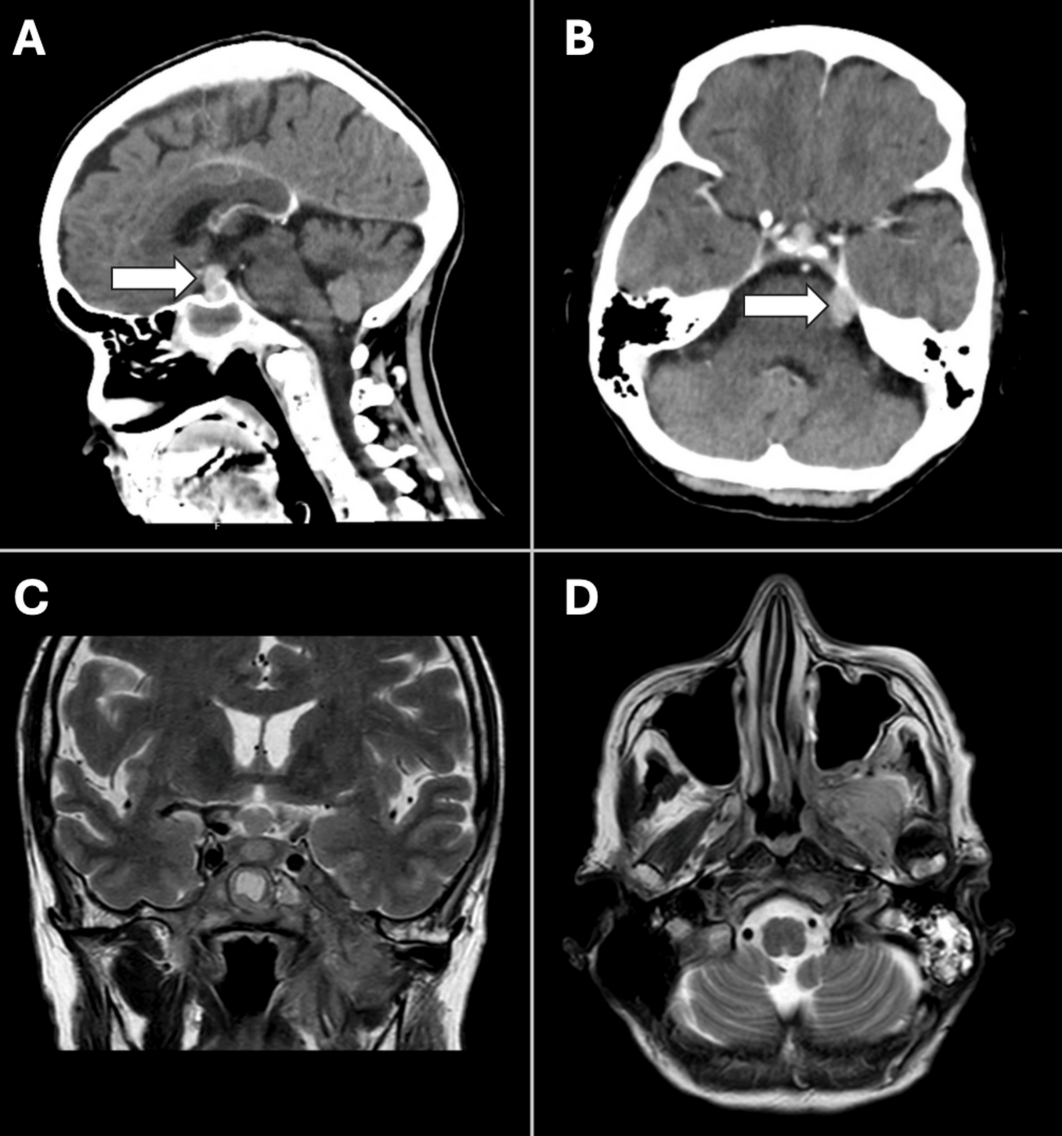
Repeat contrast-enhanced head CT scan six weeks following the initial brain imaging showing a large base of skull tumour filling the sphenoid sinus, extending superiorly to the left orbit. Significant progression in pituitary enlargement is observed, with the tumour extending into the posterior ethmoid sinuses (A). Additionally, there is a new enhancing disease in the left posterior fossa, just below the tentorium, abutting the midbrain but without causing compression (B). MRI pituitary, without contrast, T2-W images show further progression of abnormal soft tissue infiltration into the skull base, masticator space, sella, infundibulum and hypothalamus (C, D).

Unfortunately, before the haematology review, the patient experienced a fall at home. She returned to the ED, where follow-up brain imaging, six weeks after the initial MRI, revealed further disease progression. The CT imaging showed soft tissue extension into the posterior ethmoid sinuses, and new enhancing lesions were observed in the left posterior fossa, just beneath the tentorium, abutting the midbrain and breaching the dura, though not extending into the brain itself. Significant pituitary enlargement was noted, extending superiorly and encroaching upon the optic chiasm. The follow-up MRI, without contrast, demonstrated more extensive soft tissue infiltration, with skull base, masticator space, sella, infundibulum and hypothalamus extension all appearing more advanced (Figure 4).

The haematology multidisciplinary team (MDT) decided on an initial cycle of MATRIX combined chemotherapy and CD20 monoclonal antibody targeted therapy (methotrexate, cytarabine (Ara-C), thiotepa and rituximab) due to the breach of the dura and the potential involvement of the central nervous system (CNS). Future treatments will likely include alternative regimens, such as CHOP/MATRIX or R-CODOX-M/R-IVAC, with neuro-oncology expertise being consulted for further management.

## Discussion

Sinus malignancies are rare, with the sphenoid sinus being the least prevalent primary malignancy of the paranasal sinuses [5]. DLBCL is the most prevalent form of malignant lymphoma, accounting for 30%-40% of cases in adults [6]. PSBLs are exceptionally rare, and there are few case reports of those presenting with abducens nerve palsy [3,4,7,8].

In this case, the tumour was centered within the left sphenoid bone itself, with extensive soft tissue perineural extension obliterating the left pterygopalatine fossa and extending into the cavernous sinus, left orbital apex and along the posterior wall of the maxillary antrum. Rapid disease progression resulted in soft tissue extension into the posterior ethmoid sinuses, with new enhancing disease in the left posterior fossa breaching the dura at the level of the midbrain. A review of eight PSBL cases found the cavernous sinus was the most commonly affected region, and the middle skull base, subtemporal fossa, pterygopalatine fossa and sphenopetroclival region had been frequently invaded [9]. 

PSBL typically presents non-specifically and can be diagnostically challenging, as symptoms often overlap with more common pathologies such as sinus inflammation, metastatic tumours and meningiomas. A review by Marinelli et al. found that the most common presentations of PSBL included diplopia (52%), trigeminal hypoesthesia (38%), headache (29%), facial nerve weakness (25%) and subjective hearing loss (21%) [10]. B symptoms (fevers, night sweats and/or weight loss) were present in just a quarter of cases. Parosmia was not a common presentation [10]. In our case, the presentation of parosmia with a unilateral abducens nerve palsy is notable, though a review by Pesce et al. found abducens nerve palsies to be the most common cranial nerve involvement in PSBL [2]. Guan et al. present a similar case of PSBL with sphenoid, cavernous and ethmoid sinus involvements presenting with abducens nerve palsy and symptoms of sinusitis [3]. Scuotto et al. report an abducens nerve impairment due to clival PSBL with expansion toward the right petrous apex with extensive bone destruction [4]. 

Treatment is typically combined chemotherapy, though radiology alone has been used previously [10]. A common therapeutic regimen in DLBCL, including PSBL, is R-CHOP combination therapy, involving rituximab, cyclophosphamide, doxorubicin, vincristine and prednisone [11]. In this particular case, given the MRI identifying the dural breach, and possible CNS involvement, haematology MDT discussion opted for an initial cycle of MATRIX combined chemotherapy and targeted therapy (methotrexate, Ara-C, thiotepa and rituximab), which is a typical regimen for primary CNS lymphomas [12]. The literature on the use of MATRIX therapy for PSBL is scarce.

The prognosis for PSBL is variable, but this patient’s outlook is likely poorer due to the aggressive tumour spread and rapid clinical deterioration, compounded by a relatively older age of 66. Marinelli et al. found age over 60 is significantly linked to worse overall survival [10]. For patients over 60, survival rates at one, five and 10 years were 85%, 56% and 41%, respectively, compared to a 90% survival rate at 10 years for those under 60. Additionally, they found that a time to diagnosis of six months or more after symptom onset was associated with better survival, suggesting a milder disease course (five-year survival of 84% vs. 62%) [10].

## Conclusions

This case reports a rare PSBL presenting with abducens nerve palsy in a 66-year-old previously well female patient. Brain imaging revealed a large locally advanced left sphenoid tumour with extensive skull base involvement and extension into the left orbit. The diagnostic biopsy revealed a high-grade DLBCL of the germinal center type. The patient was treated initially with MATRIX combined chemotherapy and targeted therapy. 
